# FAM83H and casein kinase I regulate the organization of the keratin cytoskeleton and formation of desmosomes

**DOI:** 10.1038/srep26557

**Published:** 2016-05-25

**Authors:** Takahisa Kuga, Mitsuho Sasaki, Toshinari Mikami, Yasuo Miake, Jun Adachi, Maiko Shimizu, Youhei Saito, Minako Koura, Yasunori Takeda, Junichiro Matsuda, Takeshi Tomonaga, Yuji Nakayama

**Affiliations:** 1 Department of Biochemistry & Molecular Biology, Kyoto Pharmaceutical University, Yamashina-ku, Kyoto 607-8414, Japan; 2 Laboratory of Animal Models for Human Diseases, National Institutes of Biomedical Innovation, Health and Nutrition, Ibaraki, Osaka 567-0085, Japan; 3 Division of Anatomical and Cellular Pathology, Department of Pathology, Iwate Medical University, Shiwa-gun, Iwate 028-3694, Japan; 4 Department of Histology and Developmental Biology, Tokyo Dental College, Chiyoda-ku, Tokyo 101-0061, Japan; 5 Laboratory of Proteome Research, National Institutes of Biomedical Innovation, Health and Nutrition, Ibaraki, Osaka 567-0085, Japan

## Abstract

FAM83H is essential for the formation of dental enamel because a mutation in the FAM83H gene causes amelogenesis imperfecta (AI). We previously reported that the overexpression of FAM83H often occurs and disorganizes the keratin cytoskeleton in colorectal cancer cells. We herein show that FAM83H regulates the organization of the keratin cytoskeleton and maintains the formation of desmosomes in ameloblastoma cells. FAM83H is expressed and localized on keratin filaments in human ameloblastoma cell lines and in mouse ameloblasts and epidermal germinative cells *in vivo*. FAM83H shows preferential localization to keratin filaments around the nucleus that often extend to cell-cell junctions. Alterations in the function of FAM83H by its overexpression, knockdown, or an AI-causing truncated mutant prevent the proper organization of the keratin cytoskeleton in ameloblastoma cells. Furthermore, the AI-causing mutant prevents desmosomal proteins from being localized to cell-cell junctions. The effects of the AI-causing mutant depend on its binding to and possible inhibition of casein kinase I (CK-1). The suppression of CK-1 by its inhibitor, D4476, disorganizes the keratin cytoskeleton. Our results suggest that AI caused by the FAM83H mutation is mediated by the disorganization of the keratin cytoskeleton and subsequent disruption of desmosomes in ameloblasts.

FAM83H was originally discovered by a computational analysis of the human genomic sequence, and was then identified as a protein that plays an important role in the formation of dental enamel because a mutation in the last exon of the FAM83H gene causes amelogenesis imperfecta (AI)^1^,^2^. All AI-causing mutations of FAM83H, except for one, lead to a premature termination codon in the last exon^2^,^3^. The truncated proteins of FAM83H have been hypothesized to exert a dominant negative function that triggers AI. A previous study reported that mRNA for FAM83H was expressed in dental ameloblasts^4^, which play important roles in the entire formation process of enamel^5^. However, the mechanisms by which FAM83H regulates the formation of enamel and its truncated mutants cause AI currently remain unknown.

We recently reported the involvement of FAM83H in colorectal cancer^6^. FAM83H has been suggested to regulate the organization of the keratin cytoskeleton by recruiting CK-1α to keratin filaments in colorectal cancer cells. FAM83H was overexpressed in invading colorectal cancer cells, and, as a result, disorganized the keratin cytoskeleton. Based on these findings, we proposed that the disruption of the keratin cytoskeleton by the overexpression of FAM83H promotes the migration and invasion of colorectal cancer cells. To the best of our knowledge, apart from our previous findings, no other studies have examined the biochemical or molecular cell biological functions of FAM83H.

The keratin cytoskeleton is primarily observed in epithelial cells^7^,^8^. Keratin filaments are formed through the specific pairing of type I and II keratins. The human genome contains 28 and 26 genes that encode type I and II keratins, respectively. These keratin members are expressed in a cell type-specific manner; for example, colorectal cancer cells mainly express some of keratins 7, 8, 18, 19, and 20^7^. On the other hand, integumentary cells, including dental ameloblasts and epidermal cells, characteristically express keratins 5 and 14^7^,^9^,^10^,^11^.

Keratin filaments contribute to maintaining the mechanical stability of epithelial cells^8^,^12^,^13^. These filaments are attached to the cytoplasmic plaques of desmosomes at cell-cell junctions^14^,^15^. This attachment is required for the maintenance of desmosomes^15^,^16^,^17^. Previous studies suggested the involvement of keratins and desmosomal proteins in the formation of enamel^18^,^19^,^20^; thus, FAM83H may control the formation of enamel by regulating the organization of the keratin cytoskeleton and formation of desmosomes. In order to obtain a better understanding of the mechanisms underlying FAM83H-mutation diseases, we need to determine whether FAM83H regulates the organization of the keratin cytoskeleton in dental ameloblasts, as well as colorectal cancer cells, in spite of the different expression profiles of keratin subtypes.

In the present study, we show that FAM83H and its associated protein CK-1ε are co-localized with keratin filaments in mouse integumentary cells such as dental ameloblasts and epidermal germinative cells *in vivo*, and demonstrate that FAM83H and CK-1 regulate the organization of the keratin cytoskeleton and maintain the formation of desmosomes in human ameloblastoma cell lines. Our results suggest that AI-causing mutants of FAM83H disrupt the keratin cytoskeleton and subsequently impair the formation of desmosomes.

## Results

### FAM83H and CK-1ε are localized on keratin filaments in dental enamel cells and epidermal germinative cells *in vivo*

**Figure 1:**
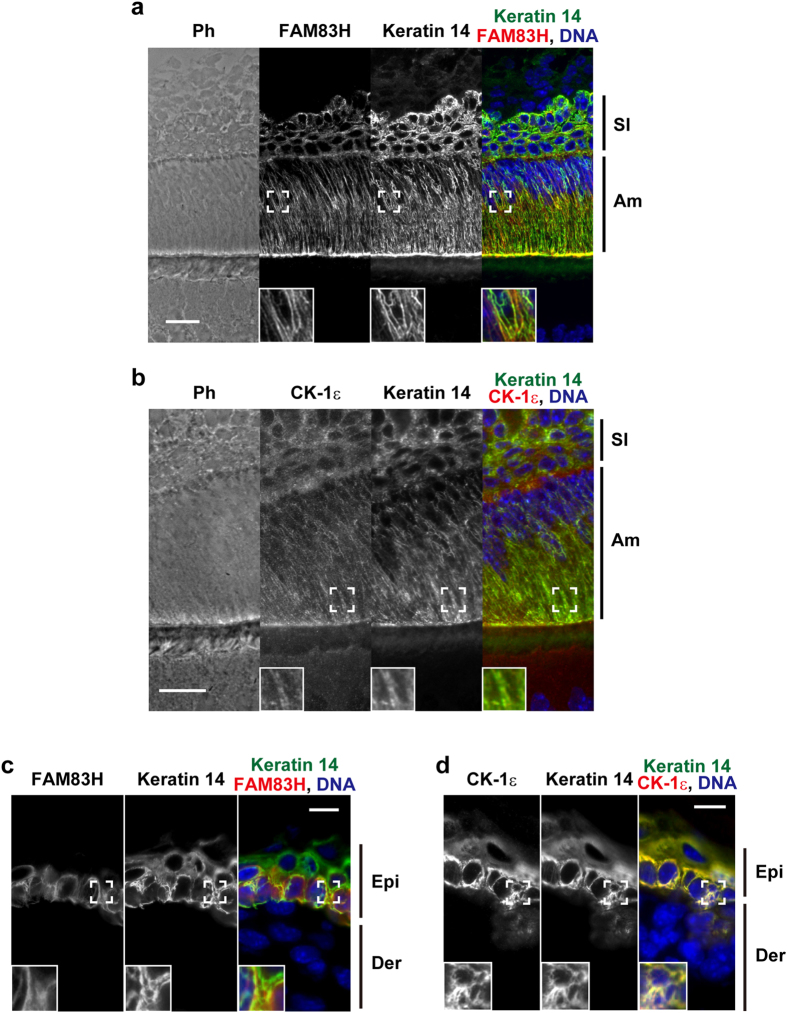
FAM83H and CK-1ε are co-localized with keratin filaments in dental enamel cells and epidermal germinative cells *in vivo*. The lower incisors (**a**,**b**) and skin (**c**,**d**) of C57BL6/NCrSlc mice on postnatal day 0 were analyzed by immunofluorescence using the indicated antibodies. Nuclei were stained using DAPI (blue). The insets are magnified images at the areas enclosed by white squares. Scale bars: 20 μm (**a**,**b**) or 10 μm (**c**,**d**). Am, ameloblasts; SI, stratum intermedium cells; Epi, epidermis; Der, dermis; Ph, phase-contrast.

In order to examine the expression and subcellular localization of FAM83H and CK-1 in dental enamel cells, we performed immunofluorescent staining of the incisors of C57BL/6NcrSlc mice using antibodies against FAM83H, CK-1 members, and keratin 14. FAM83H was expressed and co-localized on keratin filaments in ameloblasts and stratum intermedium cells (Fig. 1a). CK-1ε was also expressed and co-localized on keratin filaments in these enamel cells (Fig. 1b). CK-1α and CK-1δ were not detected, and this may have been due to these subtypes not being expressed in dental enamel cells or the antibodies against these subtypes used here not working in mice (data not shown).

Microarray analyses of human tissue samples suggested that FAM83H is also expressed in skin (http://www.ebi.ac.uk/gxa/home). Skin, as well as dental enamel organs, belongs to an integumentary tissue; thus, we tested whether FAM83H and CK-1ε are localized on keratin filaments in mouse skin. Figure 1c,d show that FAM83H and CK-1ε were expressed and co-localized with keratin filaments in epidermal germinative cells. These results imply that FAM83H and CK-1ε are involved in the organization of the keratin cytoskeleton in integumentary cells, as shown previously in colorectal cancer cells^6^.

### FAM83H is localized on keratin filaments in human ameloblastoma cells

In order to determine whether FAM83H is involved in the organization of the keratin cytoskeleton in dental enamel cells, we used the human ameloblastoma cell lines, HAM2 and HAM3, which were established from human ameloblastoma tissue^21^. These cell lines have been shown to express some marker proteins of ameloblasts^21^. The expression profiles of keratin subtypes in these cell lines were examined by next-generation sequencing. Both cell lines mainly expressed keratins 5, 6, 8, 14, 17, and 18 (Table S1).

**Figure 2:**
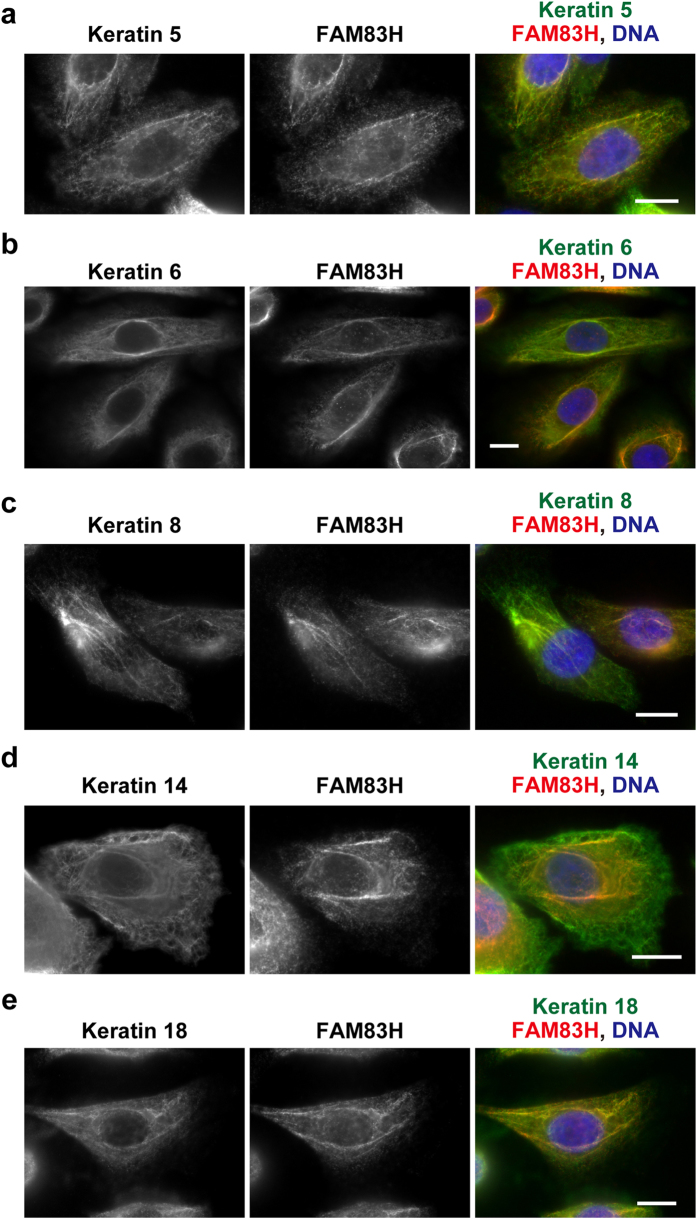
FAM83H is co-localized with keratin filaments around the nucleus in HAM2 cells. HAM2 cells were stained with the indicated antibodies. Nuclei were stained using DAPI (blue). Scale bars, 10 μm.

In an attempt to establish whether FAM83H is localized on keratin filaments in ameloblastoma cells, we performed co-immunostaining of HAM2 cells using antibodies against FAM83H and each subtype of keratin (keratins 5, 6, 8, 14, and 18). All tested keratin subtypes were largely co-localized with FAM83H (Fig. 2a–e). Keratin 17 was co-localized with keratin 8 (Fig. S1); thus, FAM83H also appears to be co-localized with filaments of keratin 17. These results suggest the involvement of FAM83H in the organization of the keratin cytoskeleton in HAM2 cells.

### FAM83H regulates the organization of the keratin cytoskeleton in ameloblastoma cells

**Figure 3:**
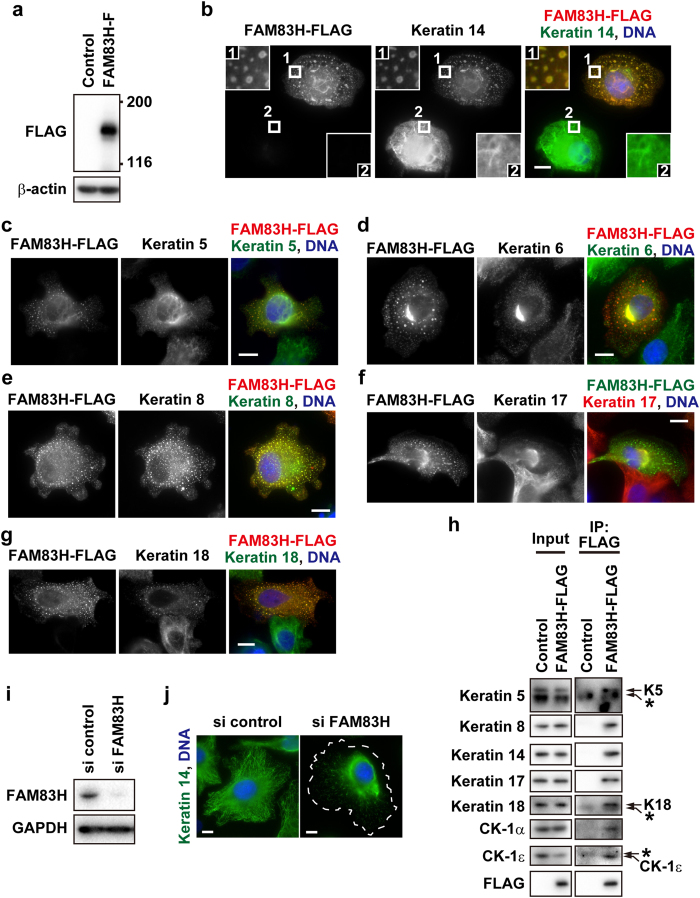
FAM83H regulates the organization of the keratin cytoskeleton in HAM2 cells. (**a,h**) HAM2 cells were transfected with FAM83H-FLAG or the control vector and analyzed by Western blotting (**a**), immunofluorescence (**b–g**), and a co-immunoprecipitation assay (**h**) using the indicated antibodies. In (**b**), insets indicate magnified images at the areas enclosed by white squares on a cell with (region 1) or without (region 2) the expression of FAM83H-FLAG. In (**h**), asterisks indicate non-specific bands because they were detected in the lanes of immunoprecipitates (IP) from not only FAM83H-FLAG-expressing cells, but also control cells. (**i,j**) HAM2 cells were transfected with FAM83H siRNA or control siRNA and analyzed by Western blotting (**i**) or immunofluorescence (**j**). In (**j**), the cell edge of a FAM83H-depleted cell is indicated by the dotted line. In (**b–g**,**j**), DNA was stained with DAPI (blue) and scale bars indicate 10 μm.

In order to assess whether FAM83H regulates the organization of the keratin cytoskeleton in ameloblastoma cells, we examined the effects of the expression levels of FAM83H on the organization of the keratin cytoskeleton using its overexpression (Fig. 3a) or knockdown (Fig. 3i). We first analyzed keratin filaments in HAM2 cells transfected with the FAK83H-FLAG vector. Immunostaining of overexpressed FAM83H-FLAG showed patterns similar to speckles or vesicles (Fig. 3b–g). The staining patterns of keratins 5, 8, 14, and 18 changed from filamentous shapes into speckle-like shapes (Fig. 3b,c,e,g; see also Fig. 2a,c,d,e), suggesting that the overexpression of FAM83H-FLAG disrupted the filaments of these keratins. These speckles of keratins clearly overlapped with the speckles of FAM83H-FLAG. The filamentous staining of keratins 6 and 17 was also severely disrupted by the overexpression of FAM83H-FLAG (Fig. 3d,f). The remaining filaments of keratins 6 and 17 around the nucleus were co-localized with FAM83H-FLAG; however, these keratins were barely detected within the speckles of FAM83H-FLAG. The co-localization of FAM83H-FLAG with keratins in HAM2 cells was supported by a co-immunoprecipitation assay showing that FAM83H-FLAG directly or indirectly interacted with keratins 5, 8, 14, 17, and 18 (Fig. 3h); however, it was not possible to assess keratin 6 because no band for keratin 6 was detected by the antibody used here in either the input or IP lanes (data not shown).

We subsequently observed keratin filaments in HAM2 cells transfected with FAM83H siRNA. Immunofluorescence for keratin 14 revealed that the depletion of FAM83H resulted in the accumulation of keratin filaments around the nucleus (Fig. 3j). Taken together, these results indicate that the aberrant expression of FAM83H prevents the proper organization of the keratin cytoskeleton in ameloblastoma cells.

### Truncated mutants of FAM83H disorganize the keratin cytoskeleton in ameloblastoma cells

**Figure 4:**
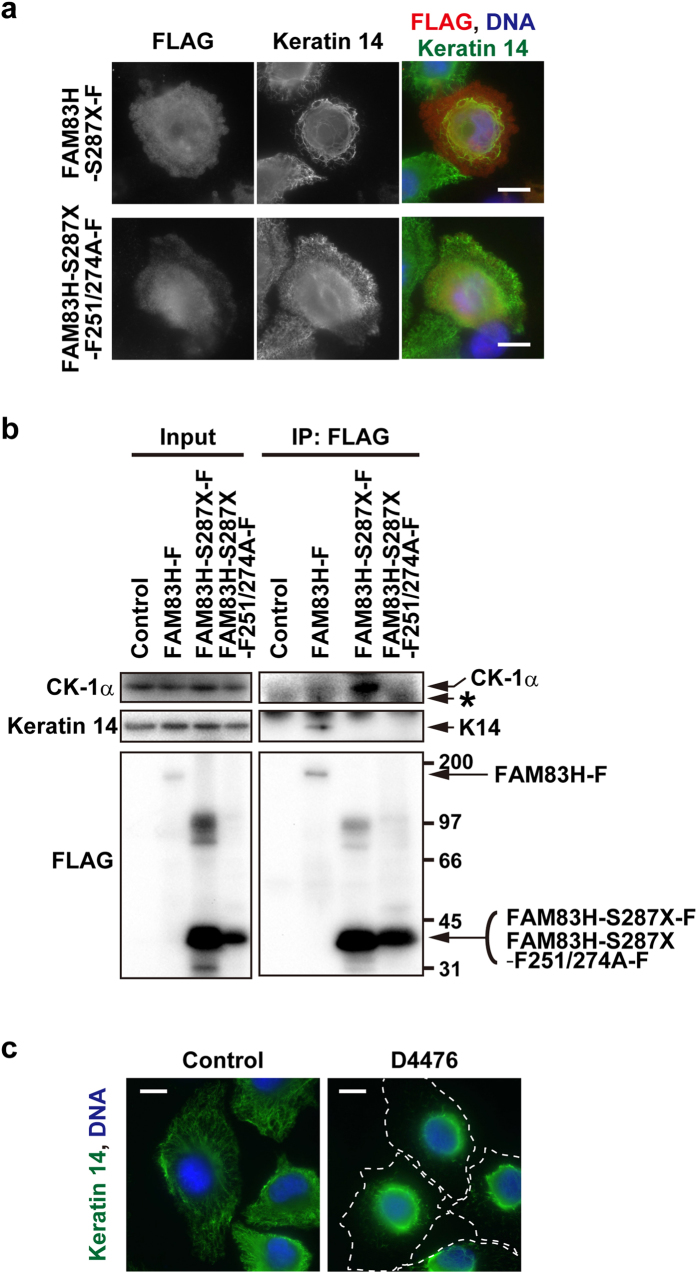
A truncated mutant of FAM83H disorganizes the keratin cytoskeleton in a manner dependent on its binding to CK-1 in HAM2 cells. (**a**) HAM2 cells were transfected with FAM83H-S287X-FLAG or FAM83H-S287X-F251/274A-FLAG and co-stained with anti-FLAG and anti-keratin 14 antibodies. (**b**) A co-immunoprecipitation assay with an anti-FLAG antibody was performed using lysates from HAM2 cells transfected with FAM83H-FLAG, FAM83H-S287X-FLAG, FAM83H-S287X-F251/274A-FLAG, or the empty vector (control). Input lysates and immunoprecipitates (IP) were analyzed by Western blotting. The positions of marker proteins (kDa) are indicated on the right side of the lower right panel. An asterisk indicates a non-specific band. (**c**) HAM2 cells were treated with 100 μM D4476, a CK-1 inhibitor, or DMSO (control) for 3 h and stained with an anti-keratin 14 antibody (green). Dotted lines indicate the edge of cells. In (**a**,**c**), DNA was visualized with DAPI (blue) and scale bars indicate 10 μm.

AI-causing truncated mutants of FAM83H have been suggested to have a dominant negative function^6^; thus, we herein tested whether these truncated mutants affect the organization of the keratin cytoskeleton in ameloblastoma cells. HAM2 cells were transfected with the vector expressing a truncated mutant of FAM83H, FAM83H-S287X-FLAG [amino acids 1-286], and were then analyzed by immunofluorescence using anti-keratin 8 and 14 antibodies. Similar to the knockdown of FAM83H, FAM83H-S287X-FLAG led to the accumulation of keratin filaments around the nucleus (Figs 4a and S2a,b). Another truncated mutant of FAM83H, FAM83H-Y297X-FLAG [amino acids 1-296], also reorganized the keratin cytoskeleton in HAM2 cells (Fig. S2c). These results suggest that truncated mutants of FAM83H disorganize the keratin cytoskeleton in ameloblastoma cells.

### A truncated mutant of FAM83H must bind to CK-1 in order to disorganize the keratin cytoskeleton in ameloblastoma cells

We previously showed that the full length of FAM83H interacts with keratins and CK-1α, resulting in the recruitment of CK-1α to keratin filaments in colorectal cancer cells^6^. In contrast, truncated mutants of FAM83H only interact with CK-1α^6^; thus, we hypothesized that truncated mutants of FAM83H sequester CK-1α from keratin filaments, leading to the dominant-negative inhibition of CK-1α. In order to test this hypothesis, we generated the FAM83H-S287X-F251/274A-FLAG mutant, which has two alanine substitutions of phenylalanines (F251 and F274) located in the consensus sequences for CK-1 binding. In order to assess the inability of FAM83H-S287X-F251/274A-FLAG to bind to CK-1 in addition to keratins, immunoprecipitates of FAM83H-S287X-F251/274A-FLAG expressed in HAM2 cells were analyzed by Western blotting using anti-keratin 14 and anti-CK-1α antibodies. The full length of FAM83H-FLAG interacted with keratin 14 and CK-1α (Figs 3h and 4b), and FAM83H-S287X-FLAG interacted with CK-1α, but not keratin 14 (Fig. 4b). FAM83H-S287X-F251/274A-FLAG interacted with neither CK-1α nor keratin 14 (Fig. 4b).

We then observed the keratin cytoskeleton in HAM2 cells transfected with FAM83H-S287X-F251/274A-FLAG. Immunofluorescence for keratin 14 showed that, in contrast to FAM83H-S287X-FLAG, FAM83H-S287X-F251/274A-FLAG did not have any effects on the organization of the keratin cytoskeleton (Fig. 4a), suggesting that truncated mutants of FAM83H must bind to CK-1 in order to function. Since the treatment of HAM2 cells with D4476, a CK-1 inhibitor, similarly disorganized the keratin cytoskeleton (Fig. 4c), these results indicate that truncated mutants of FAM83H disorganize the keratin cytoskeleton by inhibiting CK-1 in ameloblastoma cells.

### FAM83H is localized on keratin filaments that extend to cell-cell junctions

The keratin cytoskeleton plays an important role in the formation of desmosomes^13^,^15^; thus, we hypothesized that FAM83H is involved in the formation of desmosomes by regulating the organization of the keratin cytoskeleton. In order to investigate this hypothesis, we hereafter used HAM3 cells because HAM3 cells, but not HAM2 cells have the ability to establish desmosomal and adherens junctions when cultured in medium containing calcium at approximately 1.8 mM, such as IMDM or DMEM, as judged by immunostaining for the desmosomal protein, desmoplakin, and adherens junctional protein, E-cadherin (Fig. S3 and data not shown). Although the cell-cell junctions of HAM3 cells were not established in K-SFM medium containing <0.1 mM calcium, supplementation with 1.8 mM CaCl_2_ into K-SFM medium induced the formation of cell-cell junctions in HAM3 cells (Fig. S4), thereby supporting the ability of HAM3 cells to establish cell-cell junctions in a calcium-dependent manner.

**Figure 5:**
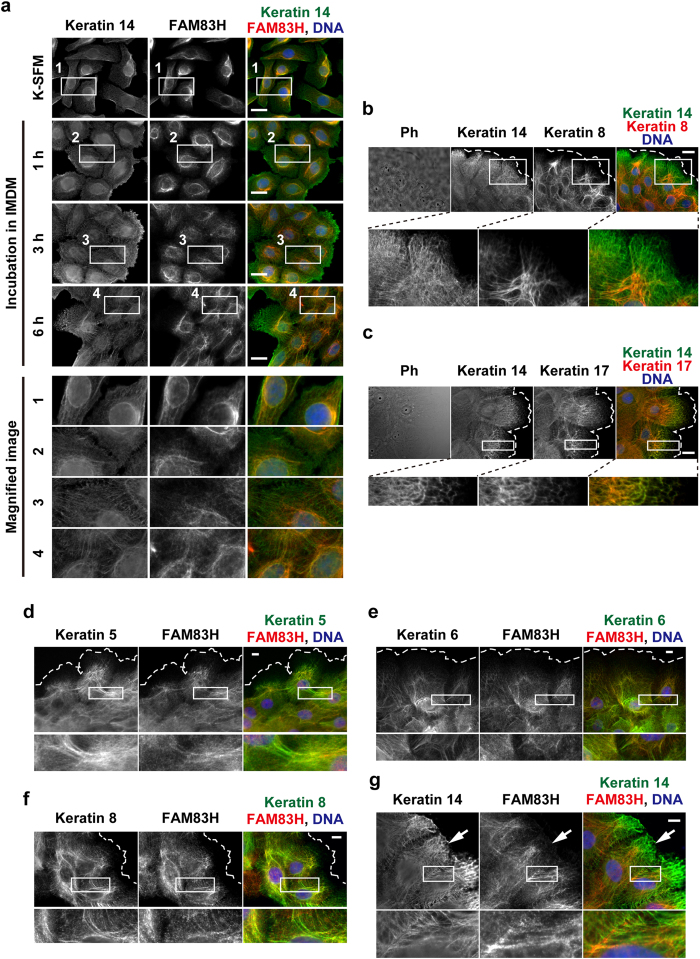
FAM83H is localized on keratin filaments extending to the cell-cell interface in HAM3 cells. (**a**) In order to induce cell-cell attachments in HAM3 cells, culture medium was replaced from K-SFM medium to IMDM medium, and cells were then incubated in IMDM medium for 1, 3, or 6 h. Cells were stained with anti-keratin 14 and anti-FAM83H. (**b**–**g**) HAM3 cells were cultured in IMDM medium for 24 h and then stained using the indicated antibodies. The edge of cell sheets was indicated by dotted lines. In (**g**), an arrow indicates the cell-spreading region. In all images, DNA was stained by DAPI (blue), the magnified images at the regions enclosed by squares are shown in the margin, and scale bars indicate 20 μm (**a–c**) or 10 μm (**d–g**). Ph, phase-contrast.

We examined changes in the morphology of keratin filaments and subcellular localization of FAM83H during the establishment of cell-cell attachments of HAM3 cells by culturing in IMDM medium. As assessed by immunostaining for keratin 14, the morphology of keratin filaments dynamically changed upon culturing in IMDM medium (Fig. 5a). Keratin filaments around the nucleus appeared to gradually extend to the cell-cell interface concomitantly with the establishment of cell-cell attachments. Keratin filaments extending to the cell-cell interface were clearly established after 6 h of culturing in IMDM medium. The subcellular localization of FAM83H also dynamically changed upon culturing in IMDM medium (Fig. 5a). Although filamentous staining of FAM83H was preferentially detected on keratin filaments around the nucleus when culturing in K-SFM medium, it expanded to the cell-cell interface concomitantly with the expansion of keratin filaments into the cell-cell interface during culturing in IMDM medium (Fig. 5a). These results suggest that FAM83H is involved in the formation of keratin filaments extending to cell-cell junctions.

We then determined which subtypes of keratins were co-localized with FAM83H in HAM3 cells cultured in IMDM medium. Although we could not perform immunostaining for keratin 18 because of its high background in HAM3 cells (data not shown), we successfully analyzed the subcellular localization of keratins 5, 6, 8, 14, and 17 by immunostaining. Filamentous staining of all tested keratin subtypes extended to the cell-cell interface (Fig. 5b–g). At the cell-spreading region, filamentous staining of keratins 5, 6, and 8 looked poor (Fig. 5b–g), but that was at least faintly detected by immunostaining using high concentrations of antibodies (Fig. S5). Co-immunostaining for FAM83H and each keratin subtype suggested that FAM83H was largely co-localized with keratins 5, 6, and 8 (Fig. 5d–f). Regarding keratin 14, FAM83H was co-localized with filaments extending to the cell-cell interface (Fig. 5g, a square), but did not or weakly co-localized with filaments extending to the cell-spreading region (Fig. 5g, an arrow). Keratin 17 was largely co-localized with keratin 14 (Fig. 5c); thus, FAM83H also appears to be co-localized with filaments of keratin 17 that extend to the cell-cell interface. These results suggest that FAM83H is localized on the bundled filaments of all tested keratin subtypes that extend to cell-cell junctions.

### A truncated mutant of FAM83H prevents desmosomal proteins from being localized to cell-cell junctions

**Figure 6:**
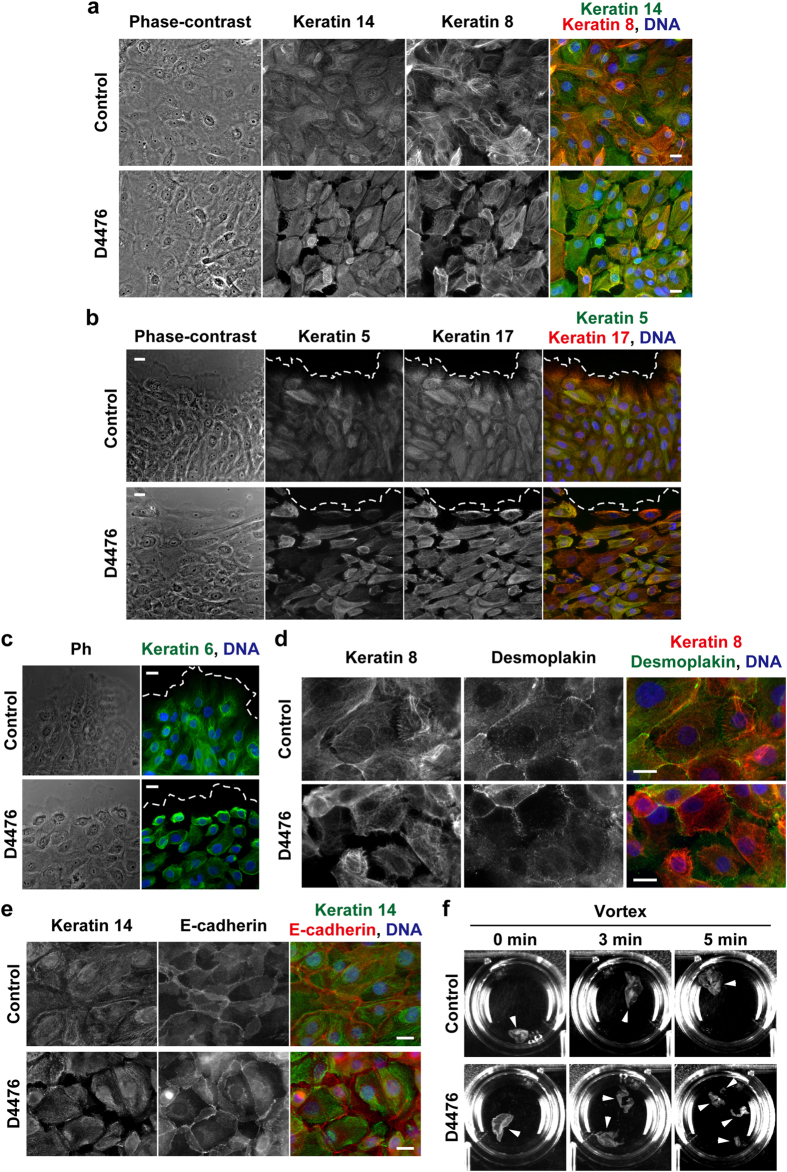
Treatment of HAM3 cells with D4476 disorganizes the keratin cytoskeleton and impairs the formation of desmosomes. HAM3 cells were cultured in IMDM medium for 24 h and then treated with 100 μM D4476 or DMSO (control) for 1 h. Cells were analyzed by immunostaining with the indicated antibodies (**a–e**) or dispase-based dissociation assay (**f**). In (**a–e**), DNA was visualized by DAPI (blue), the edge of cell sheets was indicated by dotted lines (**b,c**), and scale bars indicate 20 μm. Ph, phase-contrast. In (**f**), cell sheets were rigorously agitated on a vortex mixer for the indicated times. Arrows indicate fragments of cell sheets.

The treatment of HAM3 cells with D4476 for 1 h in IMDM medium disorganized the keratin cytoskeleton as determined based on immunostaining for keratins 5, 6, 8, 14, and 17 (Fig. 6a–c). Although cell-cell attachments appeared to be retained as judged by phase-contrast images, the extension of keratin filaments into the cell-cell interface was markedly inhibited; thus, we suspected that the formation of desmosomes in HAM3 cells may be impaired by D4476. In order to test this hypothesis, we performed immunostaining for desmoplakin in HAM3 cells treated with D4476. In cells with a keratin cytoskeleton that was severely disrupted by D4476, the localization of desmoplakin at the cell-cell interface was impaired (Fig. 6d), whereas E-cadherin was still localized to the cell-cell interface (Fig. 6e). It has been suggested that the detachment of keratin filaments with desmosomal plaques impairs intercellular adhesive strength^22^. We additionally performed a dispase-based dissociation assay on HAM3 cells treated with D4476, and the results obtained suggested that the inhibition of CK-1 decreased the intercellular adhesive strength of HAM3 cell sheets (Fig. 6f). These results suggest that CK-1 contributes to maintaining the formation of desmosomes by properly organizing the keratin filaments extending to cell-cell junctions.

**Figure 7:**
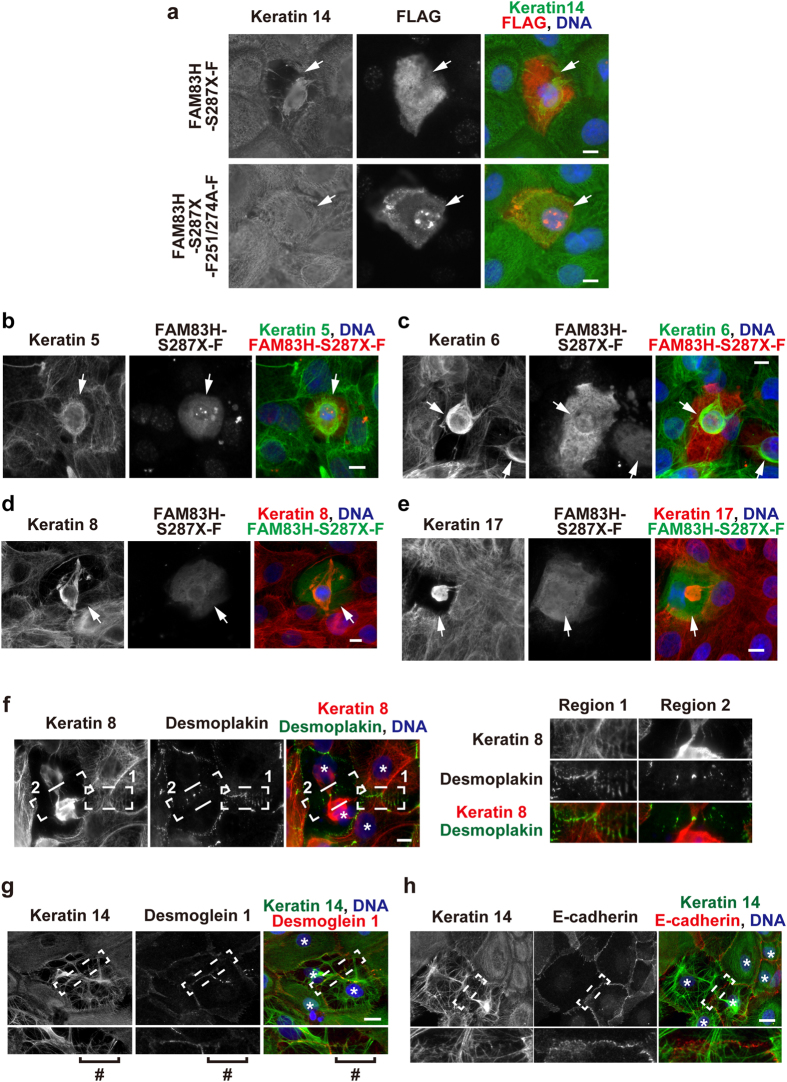
A truncated mutant of FAM83H disrupts the keratin cytoskeleton and impairs the formation of desmosomes. HAM3 cells were transfected with FAM83H-S287X-FLAG or FAM83H-S287X-F251/274A-FLAG in IMDM medium and then cultured for 24 h. Cells were stained with the indicated antibodies. DNA was visualized using DAPI (blue). Arrows indicate cells expressing a FLAG-tagged protein. Asterisks indicate the position of the nucleus. A # mark indicates the region to which keratin filaments extended less. In (**f–h**), the magnified images at the cell-cell interface enclosed by dotted lines are shown in the margin. Scale bars, 10 μm.

As observed in HAM2 cells (Fig. 4a), FAM83H-S287X-FLAG, but not FAM83H-S287X-F251/274A-FLAG severely disorganized the keratin cytoskeleton in HAM3 cells cultured in IMDM medium, as judged by immunostaining for keratins 5, 6, 8, 14, and 17 (Fig. 7a–e); thus, we assessed the formation of desmosomes in HAM3 cells transfected with FAM83H-S287X-FLAG by immunostaining for the components of desmosomes, desmoplakin, and desmoglein 1. In HAM3 cells with an intact keratin cytoskeleton, desmoplakin and desmoglein 1 were clearly localized to the cell-cell interface (Fig. 7f, region 1, 7g). In HAM3 cells showing severe disruption of the keratin cytoskeleton after transfection with FAM83H-S287X-FLAG, desmoplakin and desmoglein 1 disappeared from the cell-cell interface concomitantly with the disappearance of keratin filaments extending to the cell-cell interface (Fig. 7f, region 2; 7g, a # mark). In contrast, adherens junctions appeared to be normally established in HAM3 cells showing the disorganized keratin cytoskeleton because E-cadherin was still localized to the cell-cell interface (Fig. 7h). Taken together, these results suggest that disruption of the keratin cytoskeleton by a truncated mutant of FAM83H subsequently leads to the instability of desmosomes.

## Discussion

Although FAM83H is known to play an important role in the formation of enamel because its genetic mutations cause AI^1^,^2^,^23^,^24^, the underlying mechanism has not yet been elucidated in detail. In the present study, we showed that FAM83H and its associated protein, CK-1ε, are localized on keratin filaments in mouse dental enamel cells such as ameloblasts and stratum intermedium cells *in vivo*, and suggested that FAM83H regulates the organization of the keratin cytoskeleton and its truncated mutants disorganize the keratin cytoskeleton in a manner dependent on its CK-1 binding in human ameloblastoma cell lines. Additionally, we revealed that the disorganization of the keratin cytoskeleton by a truncated mutant of FAM83H impairs the formation of desmosomes. Since keratin proteins and desmosomal proteins are known to be involved in the formation of enamel^18^,^19^,^20^,^25^,^26^,^27^, our results suggest that AI caused by the FAM83H mutant may be mediated by the disorganization of the keratin cytoskeleton and subsequent disruption of desmosomes in dental enamel cells. The expression and localization of FAM83H and CK-1ε on keratin filaments were also observed in mouse epidermal germinative cells *in vivo*; thus, FAM83H may have the ability to regulate the organization of the keratin cytoskeleton in other integumentary cells.

We previously demonstrated that FAM83H regulates the organization of the keratin cytoskeleton in colorectal cancer cells and suggested that this regulation is mediated by recruiting CK-1α to keratin filaments^6^. The results of the present study suggest that the keratin cytoskeleton in ameloblastoma cells is regulated in a manner similar to that in colorectal cancer cells. The overexpression of FAM83H caused morphological changes in keratin filaments into the speckle-like shapes observed in ameloblastoma cells, similar to colorectal cancer cells. In both types of cells, we observed the disruption of keratin filaments into an aggregation-like morphology by the knockdown of FAM83H, the expression of its truncated mutants, and the treatment with D4476. The association of FAM83H with keratins and CK-1 was also shown by the co-immunoprecipitation assay of both types of cells. We newly revealed in the present study that FAM83H is preferentially localized on keratin filaments around the nucleus extending to cell-cell junctions. This preferential localization of FAM83H was also observed in DLD1 colorectal cancer cells (Fig. S6). In addition to colorectal cancer cells and ameloblastoma cells, FAM83H and CK-1 were localized on keratin filaments in mouse dental ameloblasts and epidermal germinative cells *in vivo* and MCF10A mammary gland cells (Fig. S7), suggesting that the keratin cytoskeleton in these cells may also be regulated by FAM83H. Given that the filaments of all keratin subtypes examined in the present study were morphologically affected by altering the function of FAM83H, FAM83H may be a common regulator of the organization of the keratin cytoskeleton in various types of cells irrespective of the expression profile of the keratin subtypes.

Our results suggest that FAM83H is involved in the formation of desmosomes, which are known to be maintained by the keratin cytoskeleton^15^,^16^,^17^. FAM83H was localized on keratin filaments extending to cell-cell junctions. Furthermore, the expression of a truncated mutant of FAM83H caused the mis-localization of the desmosomal proteins, desmoglein 1 and desmoplakin, from the cell-cell interface. In contrast, the FAM83H mutant did not cause the mis-localization of the adherens junctional protein, E-cadherin, from the cell-cell interface. The formation of adherens junctions is known to be maintained by the actin cytoskeleton^15^,^28^; thus, these results indicate that FAM83H specifically maintains the formation of desmosomes by organizing the keratin cytoskeleton.

The hypothetical mechanism of AI caused by the FAM83H mutation, as described above, has been supported by previous studies on human genetic diseases and genetically modified mice, from which it was suggested that the proper formation of the keratin cytoskeleton and desmosomes is essential for the formation of enamel. A patient with epidermolysis bullosa simplex (EBS), caused by the functional knockout of human keratin 14, exhibited mild enamel defects^18^. A female patient with compound heterozygous desmoplakin mutations exhibited enamel dysplasia^19^. Mice lacking PERP, an essential protein for stable desmosome assembly, also exhibited enamel defects^20^. In addition, in mice lacking nectin-1 or nectin-3, which function in the formation of cell-cell junctions^25^, enamel defects were observed concomitantly with the reduced formation of desmosomes in dental enamel cells^26^,^27^. In order to further substantiate our hypothesis, we are planning to generate and analyze genetically modified mice with a mutation in the FAM83H gene.

A recent study reported that FAM83H-knockout mice had a slightly scruffy coat^29^, suggesting that FAM83H plays a role in the homeostasis of skin. This phenotype may also be explained by the function of FAM83H in regulating the organization of the keratin cytoskeleton. Our results showed that FAM83H was localized on keratin filaments in epidermal germinative cells and that the knockdown of FAM83H caused the disorganization of the keratin cytoskeleton in several cell lines; therefore, the keratin cytoskeleton in epidermal germinative cells in FAM83H-knockout mice is expected to be disorganized. If this is the case, the scruffy coat may be a plausible phenotype because genetic abnormalities in keratins 5 and 14 are well-known to cause skin diseases^30^,^31^,^32^,^33^,^34^.

FAM83H appears to interact with multiple isoformes of CK-1. In the present study, co-immunoprecipitation assay showed that FAM83H interacts with CK-1α and ε. Previous interactome analyses suggested that CK-1δ may also be an interacting protein of FAM83H^6^,^35^. On the other hand, CK-1γ1, 2, and 3 might not interact with FAM83H. In contrast to CK-1α, δ, and ε, the CK-1γ isoforms were not identified by the proteomic analysis of co-immunoprecipitates with FAM83H-FLAG expressed in HCT116 cells^6^, although the CK-1γ isoformes have been suggested to be expressed in HCT116 cells^36^. Multiple isoforms of CK-1 may play a redundant role in the organization of the keratin cytoskeleton.

Further studies are needed in order to determine whether CK-1 phosphorylates keratin proteins and if this phosphorylation controls the organization of the keratin cytoskeleton. In an attempt to obtain an insight into this issue, we performed a phospho-proteomic analysis of HAM3 cells treated with D4476. Phosphorylation levels at several Ser/Thr sites in several keratin subtypes were suggested to be altered by D4476 (Table S2). Some of the phosphorylation sites were matched with the consensus sequences for the CK-1 substrates (pS/pT-X-X-S/T or D-X-X-S/T; the underlined residues refer to the target sites, pS/pT refers to a phospho-serine or phospho-threonine)^37^. Our proteomic analysis also suggested that the phosphorylation of desmoplakin may be altered by D4476 (Table S2). To date, we have confirmed by Western blotting and immunofluorescence that phosphorylation at least at Ser23 of keratin 8 was suppressed by the treatment of HAM3 cells with D4476 (Fig. S8). In future studies, we will determine the CK-1-phosphorylation sites of keratins responsible for the reorganization of the keratin cytoskeleton.

In conclusion, the present study demonstrated that FAM83H plays an important role in the organization of the keratin cytoskeleton and formation of desmosomes in ameloblastoma cells. Based on these results, we propose a possible mechanism by which the FAM83H mutation causes AI. In addition, our present results and previous findings suggest that FAM83H is a common regulator of the keratin cytoskeleton in various types of cells irrespective of the expression profile of the keratin subtype.

## Methods

### Cell culture and transfection

HAM2 and HAM3 ameloblastoma cell lines were established from a human ameloblastoma tissue, as described previously^21^. HAM cell lines were maintained at 37 °C in 5% CO_2_ in keratinocyte-SFM medium (K-SFM; Life Technologies, Carlsbad, CA, USA). DLD1 colorectal cancer cells were purchased from ATCC (Manassas, VA, USA) and cultured at 37 °C in 5% CO_2_ in DMEM medium (Nissui Pharmaceutical, Tokyo, Japan) supplemented with 5% FBS (AusGeneX, Molendinar, QLD, Australia). MCF10A mammary gland cells were obtained from the Barbara Ann Karmanos Cancer Institute Cell Line Resource (Detroit, MI, USA) and cultured at 37 °C in 5% CO_2_ in IMDM medium (Life Technologies) supplemented with 10% FBS (Life Technologies). Plasmid DNAs and siRNAs were transfected using Lipofectamine 3000 and Lipofectamine RNAiMAX, respectively (Life Technologies). The transfection of HAM cell lines was performed in IMDM medium supplemented with 10% FBS because K-SFM medium was not suitable for the transfection reaction. D4476, a CK-1 inhibitor, was used at a concentration of 100 μM (Abcam, Cambridge, UK).

### Antibodies

The following antibodies were purchased: anti-FAM83H (HPA024604; Sigma-Aldrich, St Louis, MO, USA), anti-keratin 5 (XM26; Thermo Scientific, Fremont, CA, USA), anti-keratin 6 (LHK6; Santa Cruz Biotechnology, Santa Cruz, CA, USA), anti-keratin 8 (TS1; Thermo Scientific; EP1628Y, Epitomics, Burlingame, CA, USA), anti-keratin 14 (LL002; Thermo Scientific), anti-keratin 17 (D12E5; Cell Signaling Technology, Beverly, MA, USA), anti-keratin 18 (DC10; Thermo Scientific), anti-FLAG (M2; Sigma-Aldrich, A00170; GenScript, Piscataway, NJ, USA), anti-CK-1α (C-19; Santa Cruz Biotechnology), anti-CK-1ε (HPA026288; Sigma-Aldrich), anti-CK-1δ (R-19; Santa Cruz Biotechnology), anti-desmoglein 1 (H-290; Santa Cruz Biotechnology), anti-E-cadherin (24E10; Cell Signaling Technology), anti-β-actin (AC-40; Sigma-Aldrich), anti-GAPDH (GT239; GeneTex), anti-phospho-keratin 8 Ser 23 (EP1629Y; Abcam), anti-phospho-keratin 8 Ser 431 (EP1630; Epitomics), anti-phospho-keratin 8 Ser 73 (E431–2; Abcam), and anti-desmoplakin 1&2 (cocktail of clones DP1&2–2.15, DP1–2.17, and DP1&2–2.20; Progen, Heidelberg, Germany) antibodies. Alexa Fluor 488 and 594 donkey anti-mouse IgG, Alexa Fluor 488, 555, and 594 donkey anti-rabbit IgG, and Alexa Fluor 488 and 555 donkey anti-goat IgG antibodies were used for immunofluorescence (Life Technologies). HRP-conjugated horse anti-mouse IgG (Cell Signaling Technology), donkey anti-rabbit IgG (GE Healthcare, Little Chalfont, UK), and bovine anti-goat IgG (Jackson ImmunoResearch, West Grove, PA, USA) antibodies were used for Western blotting.

### Plasmid DNA and siRNA

Plasmids encoding human FAM83H-FLAG and FAM83H-S287X-FLAG were generated in a previous study^6^. In order to generate the FAM83H-S287X-F251/274A-FLAG-expressing plasmid, we first generated the FAM83H-F251/274A-FLAG-encoding plasmid using a PrimeSTAR Mutagenesis Basal Kit (Takara Bio, Shiga, Japan). We performed a PCR reaction using the FAM83H-F274A-FLAG vector as the PCR template^6^ and a primer set (the forward primer 5′-TGGTCCGCTGAGAAGATCCACCGCAGC-3′ and reverse primer 5′-CTTCTCAGCGGACCACATGAAGCTGTA-3′). The region corresponding to 1-286 amino acids of FAM83H-F251/274A-FLAG was then amplified by PCR using the forward primer 5′-ATAGAATTCAACATGGCCCGTCGCTCTCAGAG-3′ and reverse primer 5′-ATAGGATCCGGGCACAAGCGGCTCGGACTG-3′, and then cloned into the p3XFLAG-CMV-14 vector. FAM83H siRNA (FAM83H-HSS138852) and control siRNA (Medium GC Duplex #2) were purchased from Life Technologies.

### Mice

C57BL6/NCrSlc mice were purchased from Japan SLC Inc. (Hamamatsu, Japan) and housed under specific pathogen-free conditions. The experimental mouse protocols were approved by the Ethics Committee at the National Institutes of Biomedical Innovation, Health and Nutrition (assigned No. DS26-36). All experiments using mice were conducted in accordance with the guidelines for animal experiments of the National Institutes of Biomedical Innovation, Health and Nutrition.

In order to prepare tissue sections for immunofluorescence, mice on postnatal day 0 were sacrificed and their mandibular regions or skin were collected. Tissue samples were embedded in OCT compound (Sakura Finetek, Tokyo, Japan), snap frozen in liquid nitrogen, and cut into 5-μm-thick sections using a cryostat (Hyrax C50; Carl Zeiss, Jena, Germany).

### Protein extraction, immunoprecipitation, and Western blotting

In order to extract whole cellular proteins, cells were directly lysed in SDS-PAGE sample buffer, as described previously^38^. The preparation of cell lysates for immunoprecipitation (IP lysates) was performed as described previously^6^. Briefly, cells were suspended in PBS containing 1% Triton X-100 (Nacalai Tesque, Kyoto, Japan), 0.5 mM PMSF (Nacalai Tesque), 2 μg/ml aprotinin (Seikagaku Corporation, Tokyo, Japan), 0.8 μg/ml pepstatin (Wako Pure Chemical Industries, Osaka, Japan), 2 μg/ml leupeptin (Wako Pure Chemical Industries), 10 mM β-glycerophosphate (Sigma-Aldrich), and 1 mM sodium orthovanadate (Wako Pure Chemical Industries), and then homogenized by sonication (BIORUPTOR, COSMO BIO, Tokyo, Japan). After centrifugation at 100,000 × *g* for 30 min, the supernatant was collected. Immunoprecipitation was performed using the anti-FLAG antibody (M2) cross-linked to Protein G Dynabeads (Life Technologies) by dimethyl pimelimidate dihydrochloride (Nacalai Tesque). IP lysates were reacted with antibody-coated Dynabeads at 4 °C for 3 h, and the absorbed proteins were eluted with SDS-PAGE sample buffer without reducing agents. Western blotting was performed using a chemiluminescence detection system (Chemi-Lumi One L, Super, or Ultra; Nacalai Tesque). Images were obtained with ChemiDoc XRS + (Bio-Rad, Richmond, CA, USA) and processed with Photoshop CS5 (Adobe, San Jose, CA, USA).

### Immunofluorescence

Immunofluorescence was performed as described previously^39^. Briefly, cultured cells or tissue sections were fixed with MeOH at −20 °C for 2 min, blocked using Blocking One (Nacalai Tesque) on ice, and sequentially incubated with appropriate primary and secondary antibodies at room temperature. DNA was stained with 100 ng/ml 4′-6-diamidino-2-phenylindole (DAPI; Sigma-Aldrich), and stained samples were viewed under an IX83 fluorescence microscopy (Olympus, Tokyo, Japan). The objective lenses were UPlan FL N 10×/0.30 or 40×/0.75 and PlanApo N 60×/1.42. Composite figures were prepared using Photoshop CS5.

### Dispase-based dissociation assay

HAM3 cells were seeded on 24-well dishes and cultured in IMDM medium for 24 h. At 100% confluency, cells were treated with 100 μM D4476 or DMSO for 1 h. In order to release monolayers from dishes, cells were incubated in IMDM medium containing 500 U/mL dispase (Godo Shusei, Tokyo, Japan) in addition to 100 μM D4476 or DMSO for 40 min. The monolayers obtained together with medium were transferred into 1.5-mL plastic tubes and rigorously agitated at the maximum speed of a vortex mixer (Vortex-Genie 2; Scientific Industries, Bohemia, NY, USA) for 3 or 5 min.

### Phospho-proteomic analysis

HAM3 cells cultured in K-SFM medium were treated with 100 μM D4476 or DMSO (control) for 1 h, and proteins were then extracted and proteolytically digested using a phase-transfer surfactant protocol, as described previously^40^. Phosphopeptides were enriched from 200 μg of digested peptides using immobilized Fe (III) affinity chromatography (IMAC), as described previously^40^. Phosphopeptides were analyzed by a Q Exactive mass spectrometry equipped with an Ultimate 3000 Nano LC system (Thermo Scientific) and an HTC-PAL autosampler (CTC Analytics, Zwingen Switzerland), as described priviously^41^. The identification and quantitation of phosphopeptides were performed using MaxQuant 1.3.0.5 supported by the Andromeda search engine for peptide identification^42^ and the UniProt human data base (release 2011_11), as described previously^41^. Phosphopeptides were accepted with a false discovery rate of <0.01, which was calculated using the reverse database. Quantitation was performed by the label-free quantitation mode.

### Next-generation sequencing

Next-generation sequencing was performed as described previously^43^. Briefly, total RNA was collected using an RNeasy Mini Kit (Qiagen Inc., Valencia, CA, USA) and the RNA sequencing library was prepared with a TruSeq RNA Sample Prep Kit (Illumina, San Diego, CA, USA). Paired-end 100-bp sequencing was carried out on a HiSeq 2500 instrument (Illumina). After a successful sequencing reaction, Illumina FASTQ reads were mapped to the human reference genome (hg19) using Bowtie^44^ and TopHat^45^. Aligned reads were converted to binary-sequence alignment MAP file format, sorted, and indexed using SAMtools^46^. Assembling transcripts with reference to the human reference genome (hg19) and an estimation of their abundances were performed using Cufflinks^47^. The gene analysis software was run using Diagno Linux OS (Iwate Medical University, Morioka, Iwate, Japan).

## Additional Information

**How to cite this article**: Kuga, T. *et al.* FAM83H and casein kinase I regulate the organization of the keratin cytoskeleton and formation of desmosomes. *Sci. Rep.* **6**, 26557; doi: 10.1038/srep26557 (2016).

## Supplementary Material

Supplementary Information

Supplementary Dataset
