# Supplementary material for: FAM83H and casein kinase I regulate the organization of the keratin cytoskeleton and formation of desmosomes
Source: Sci Rep. 2016 May 25;6:26557. doi: 10.1038/srep26557 (PMC4879633; doi:10.1038/srep26557)
Supplement: Supplementary Information [file srep26557-s1.pdf]

FAM83H and casein kinase I regulate the organization of the keratin cytoskeleton and formation of desmosomes

Takahisa Kuga, Mitsuho Sasaki, Toshinari Mikami, Yasuo Miake, Jun Adachi, Maiko Shimizu, Youhei Saito, Minako Koura, Yasunori Takeda, Junichiro Matsuda, Takeshi Tomonaga, and Yuji Nakayama

Supplementary figure legends

Fig. S1 The subcellular localization of keratins 8 and 17 in HAM2 cells.

HAM2 cells were stained with anti-keratin 8 and anti-keratin 18 antibodies. DNA was visualized using DAPI (blue). Scale bar, 10 μm.

Fig. S2 Different truncated mutants of FAM83H disorganize the keratin cytoskeleton in HAM2 cells.

HAM2 cells were transfected with FAM83H-S287X-FLAG (a, b) or FAM83H-Y297X-FLAG (c) and stained with anti-FLAG and anti-keratin 14 (a, c) or 8 (b) antibodies. DNA was visualized using DAPI (blue). Arrows indicate a cell expressing a FLAG-tagged protein. Scale bars, 10 μm.

Fig. S3 HAM3 cells establish cell-cell junctions when cultured in IMDM medium.

HAM3 cells were cultured in the maintenance medium, K-SFM, or in IMDM medium for 24 h. Cells were then stained with the indicated antibodies. DNA was visualized using DAPI (blue). Scale bars, 20 μm.

Fig. S4 HAM3 cells establish cell-cell junctions when treated with 1.8 mM $CaCl_2$.

HAM2 (a) and HAM3 (b) cells were cultured in K-SFM medium supplemented with 1.8 mM $CaCl_2$ or nothing for 24 h. Cells were then stained with the indicated antibodies. DNA was visualized using DAPI (blue). The areas enclosed by squares were magnified at the margin. Scale bars, 20 μm.

Fig. S5 Keratins 5, 6, and 8 are present at the cell-spreading region in HAM3 cells.

HAM3 cells were cultured in IMDM medium for 24 h and then stained with an anti-keratin 5,

6, or 8 antibody at low dilution (1:50). DNA was visualized using DAPI (blue). The edge of cell sheets was indicated by dotted lines. The magnified images at the regions enclosed by squares are shown in the margin. Scale bars, 20 μm.

Fig. S6 Immunofluorescence of DLD1 cells.

DLD1 cells were stained with the indicated antibodies. DNA was visualized using DAPI (blue). The areas enclosed by squares were magnified at the margin. The dotted lines indicate the edge of cell masses. Ph, phase-contrast images. Scale bars, 20 μm. Keratins 8 and 18 were largely co-localized with each other (a). FAM83H and CK-1α and ε were co-localized with keratin filaments around the nucleus (b-d).

Fig. S7 Immunofluorescence of MCF10A mammary gland cells.

MCF10A cells were analyzed by immunofluorescence using the indicated antibodies. DNA was visualized with DAPI (blue). Insets indicate the magnified images at the areas enclosed by squares. Scale bars, 10 μm. FAM83H and CK-1ε were co-localized with keratin filaments.

Fig. S8 Phosphorylation at Ser 23 of keratin 8 is suppressed by the inhibition of CK-1.

HAM3 cells cultured in K-SFM medium (a) or IMDM medium for 24 h (b) were treated with 100 μM D4476 or DMSO (control) for 1 h, and then analyzed by Western blotting (a) or immunofluorescence (b) using the indicated antibodies. In (b), DNA was stained with DAPI (blue) and scale bars indicate 10 μm. Phosphorylation at Ser 23, but not Ser 73 or Ser 431, of keratin 8 was suppressed by the treatment with D4476. The serine residues of keratin 8 are numbered excluding the first methionine.

Supplementary tables

Table S1 Expression profiles of keratin subtypes in HAM2 and HAM3 cells.

HAM2 and HAM3 were analyzed by next-generation sequencing, as described in the Methods section. FPKM (Fragments Per Kilobase Million) of keratin subtypes were shown. These data suggest that HAM2 and HAM3 cells both mainly express keratins 5, 6, 8, 14, 17, and 18.

Table S2 Identified phosphorylation sites of keratins and desmoplakin.

HAM3 cells were treated with 100 μM D4476 or DMSO (control) for 1 h and then analyzed by phospho-proteomic techniques, as described in the Methods section. As based on the peak intensity, some cases of phosphorylation were suggested to be suppressed (blue) or enhanced (red) by the D4476 treatment.

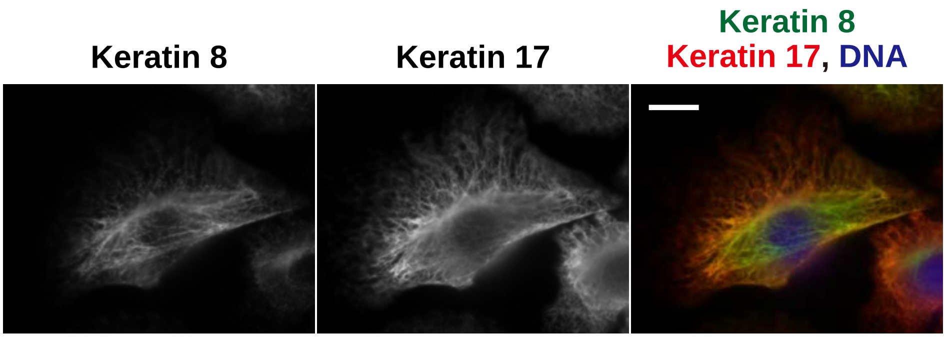

Figure S1

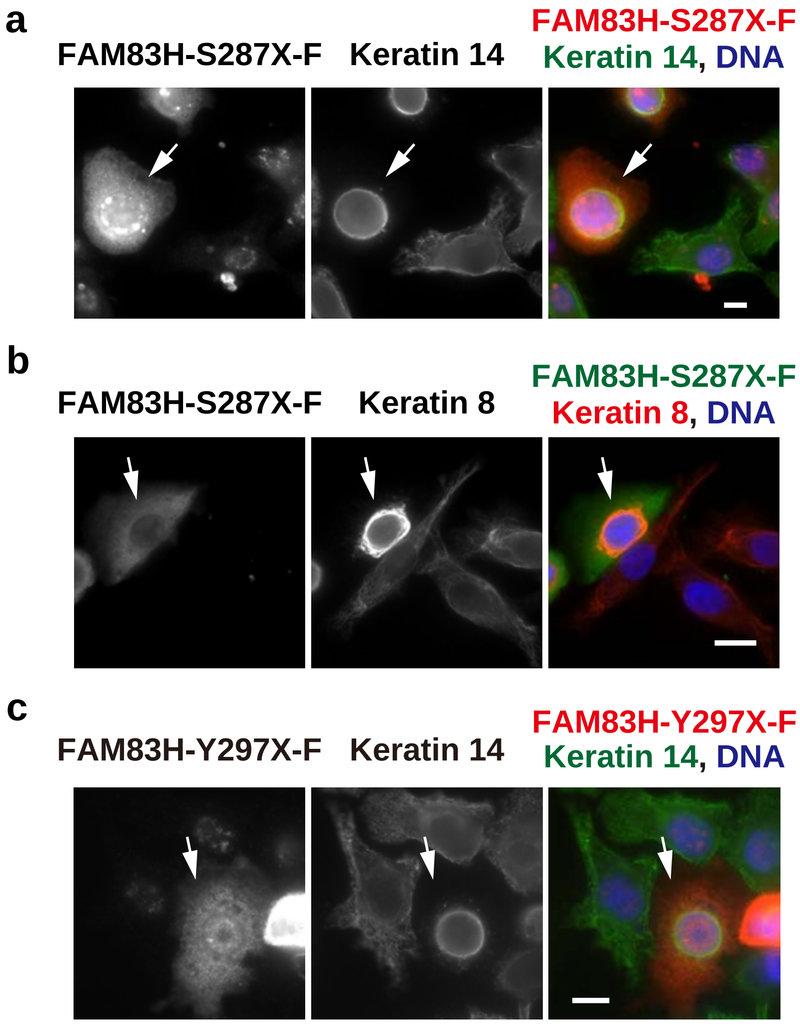
a
FAM83H-S287X-F
Keratin 14
FAM83H-S287X-F
Keratin 14, DNA
b
FAM83H-S287X-F
Keratin 8
FAM83H-S287X-F
Keratin 8, DNA
c
FAM83H-Y297X-F
Keratin 14
FAM83H-Y297X-F
Keratin 14, DNA

Figure S2

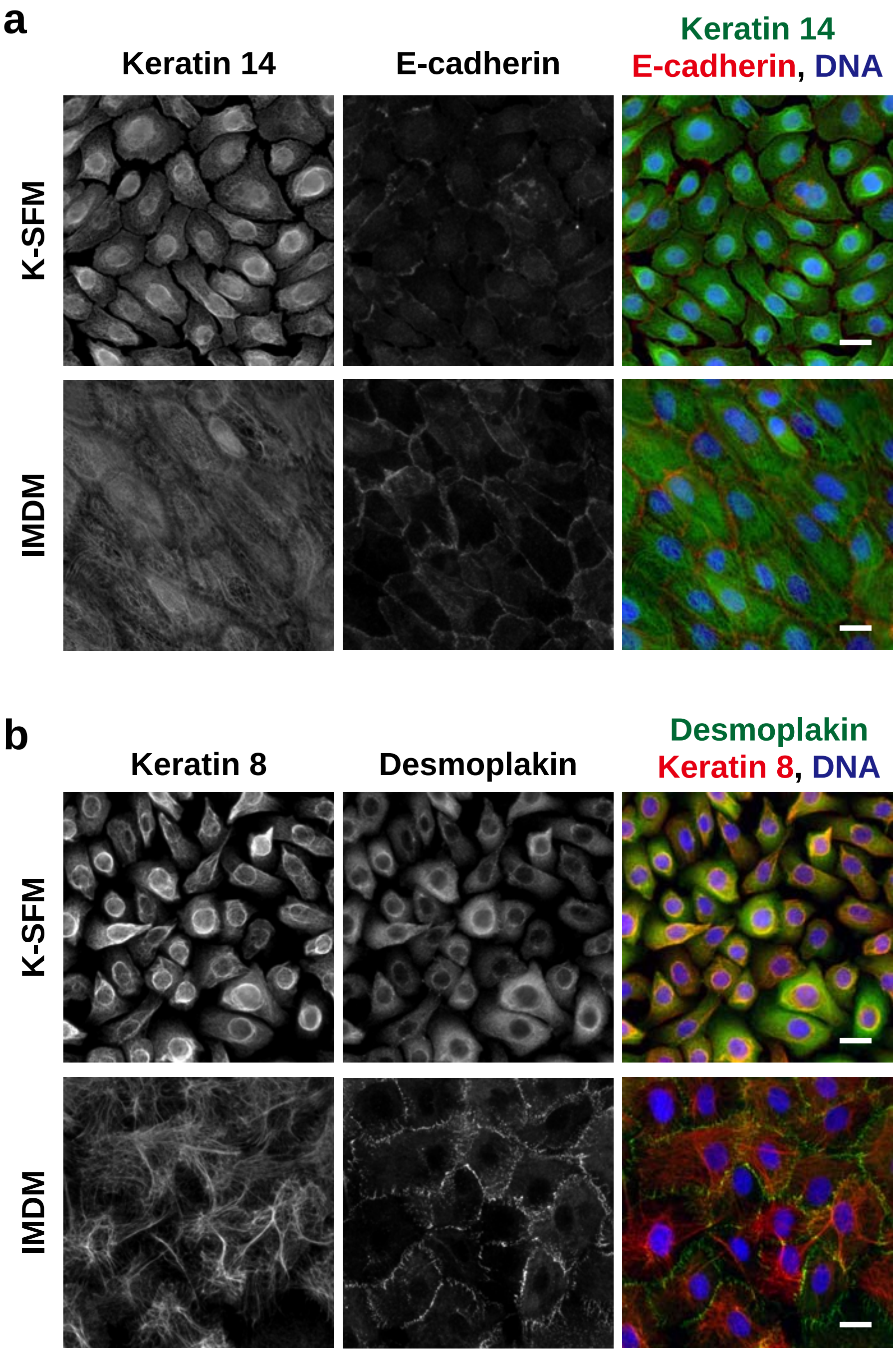
a
Keratin 14
E-cadherin
Keratin 14
E-cadherin, DNA
K-SFM
IMDM
b
Keratin 8
Desmoplakin
Desmoplakin
Keratin 8, DNA
K-SFM
IMDM

Figure S3

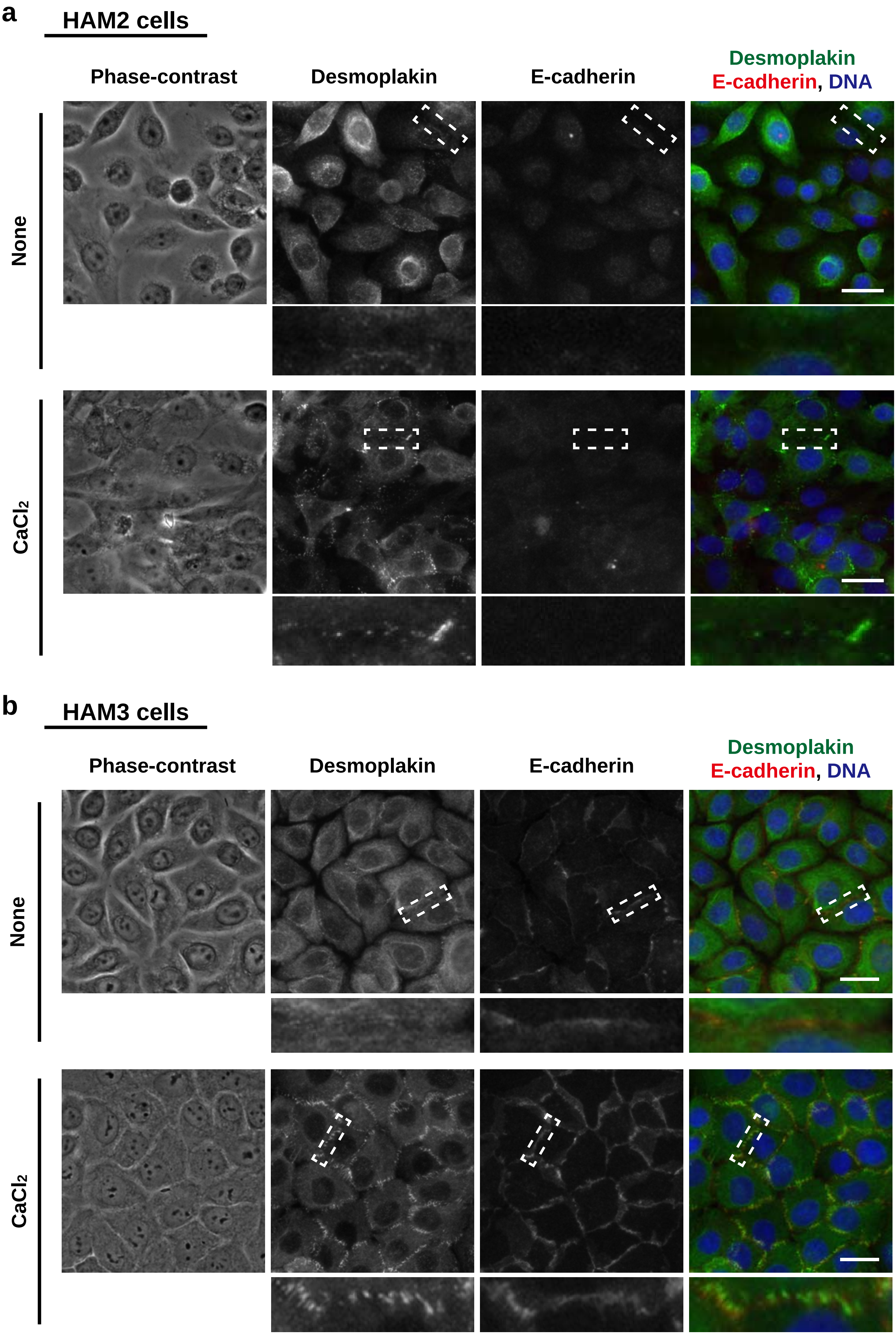

a
HAM2 cells
Phase-contrast
Desmoplakin
E-cadherin
Desmoplakin
E-cadherin, DNA
None
CaCl2
b
HAM3 cells
Phase-contrast
Desmoplakin
E-cadherin
Desmoplakin
E-cadherin, DNA
None
CaCl2

Figure S4

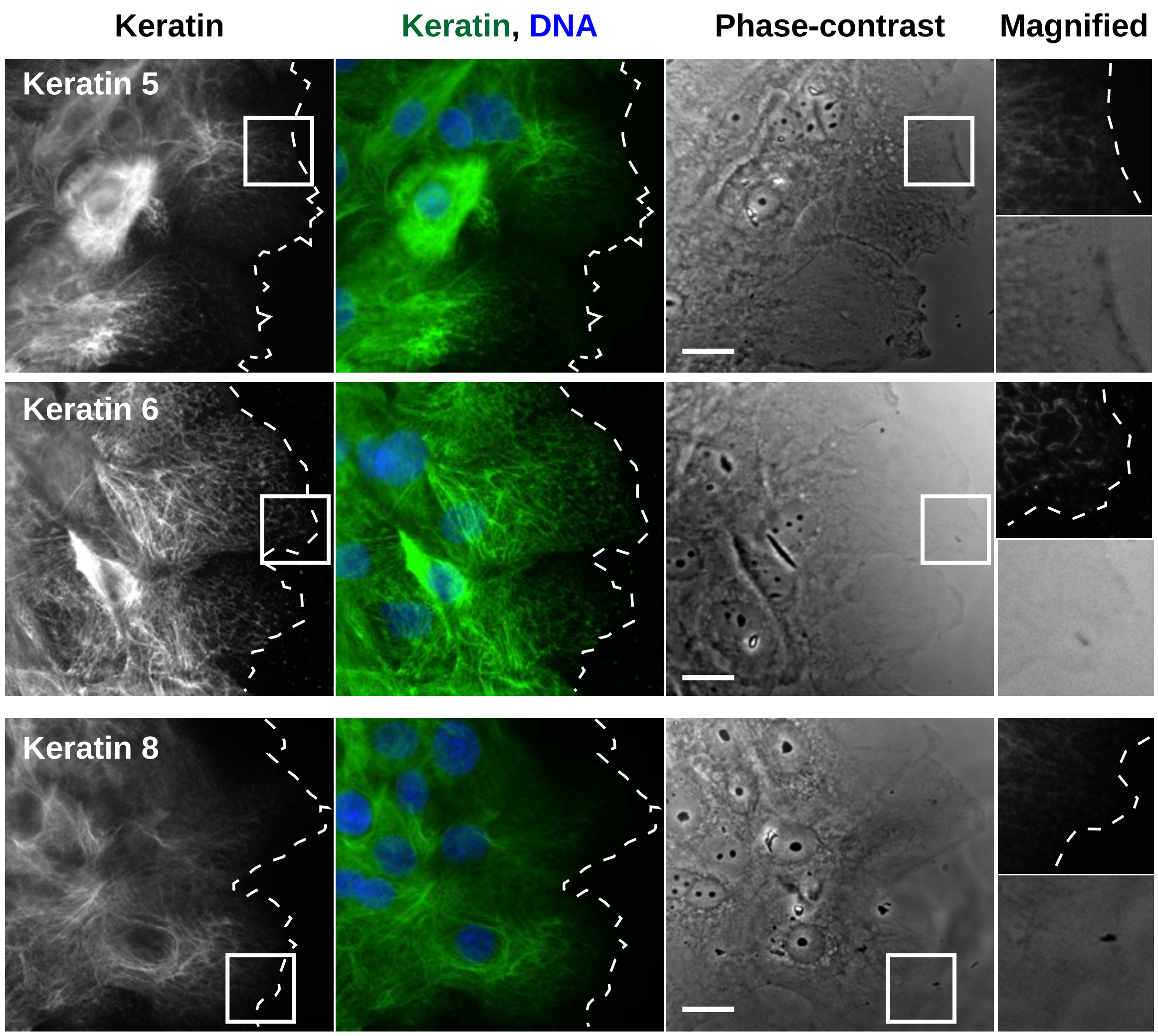
Keratin
Keratin, DNA
Phase-contrast
Magnified
Keratin 5
Keratin 6
Keratin 8

Figure S5

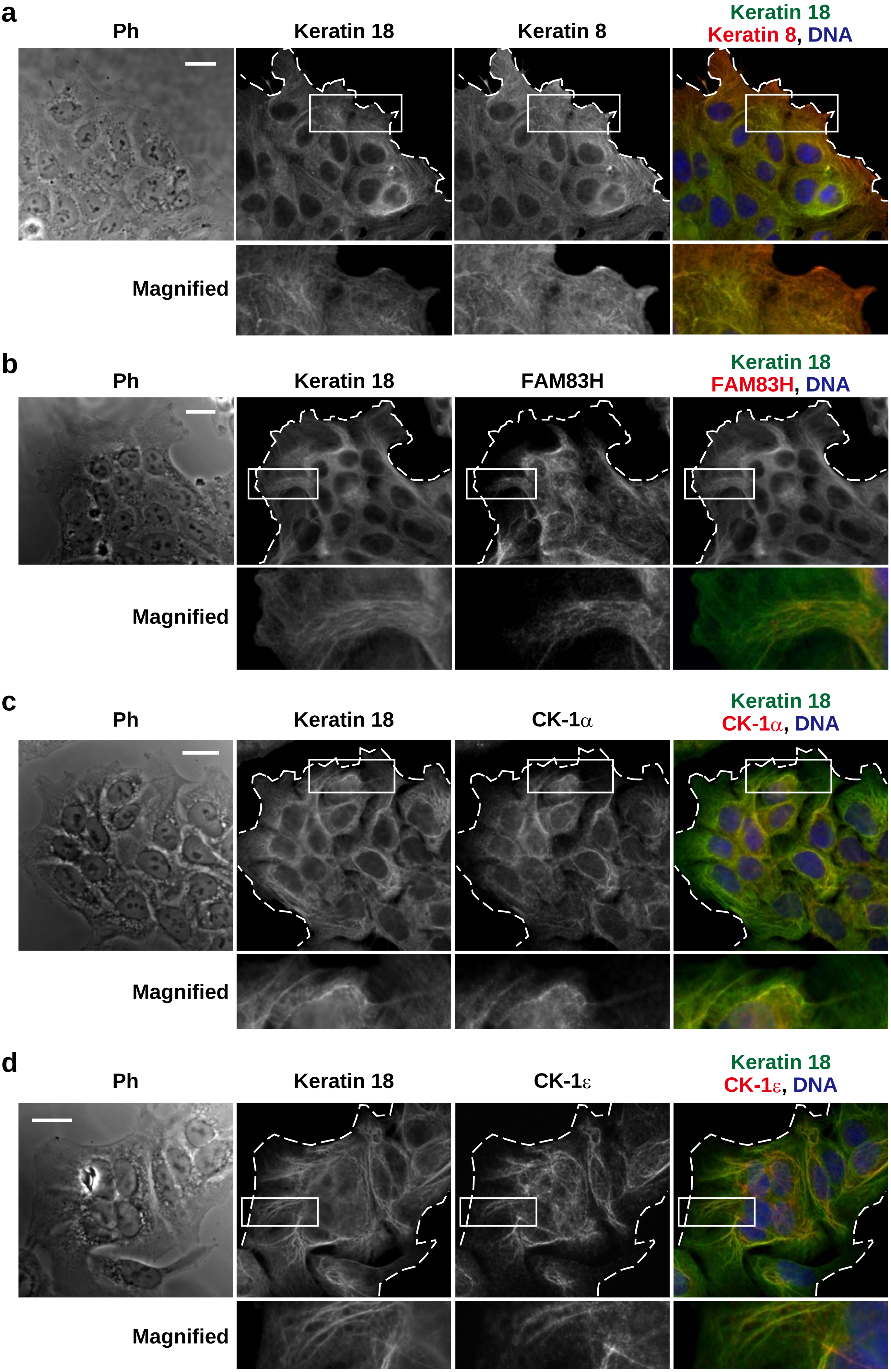
a
Ph
Keratin 18
Keratin 8
Keratin 18
Keratin 8, DNA
Magnified
b
Ph
Keratin 18
FAM83H
Keratin 18
FAM83H, DNA
Magnified
c
Ph
Keratin 18
CK-1α
Keratin 18
CK-1α, DNA
Magnified
d
Ph
Keratin 18
CK-1ε
Keratin 18
CK-1ε, DNA
Magnified

Figure S6

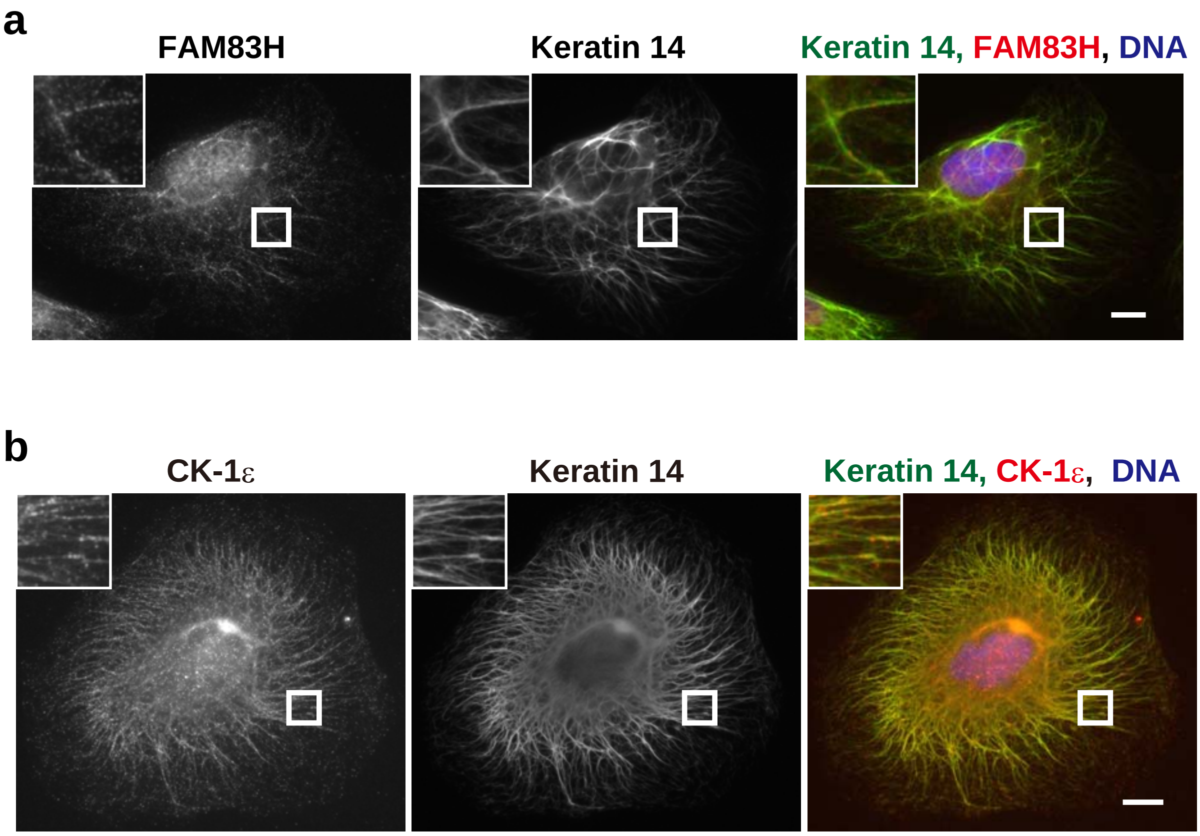
a
FAM83H
Keratin 14
Keratin 14, FAM83H, DNA
b
CK-1ε
Keratin 14
Keratin 14, CK-1ε, DNA

Figure S7

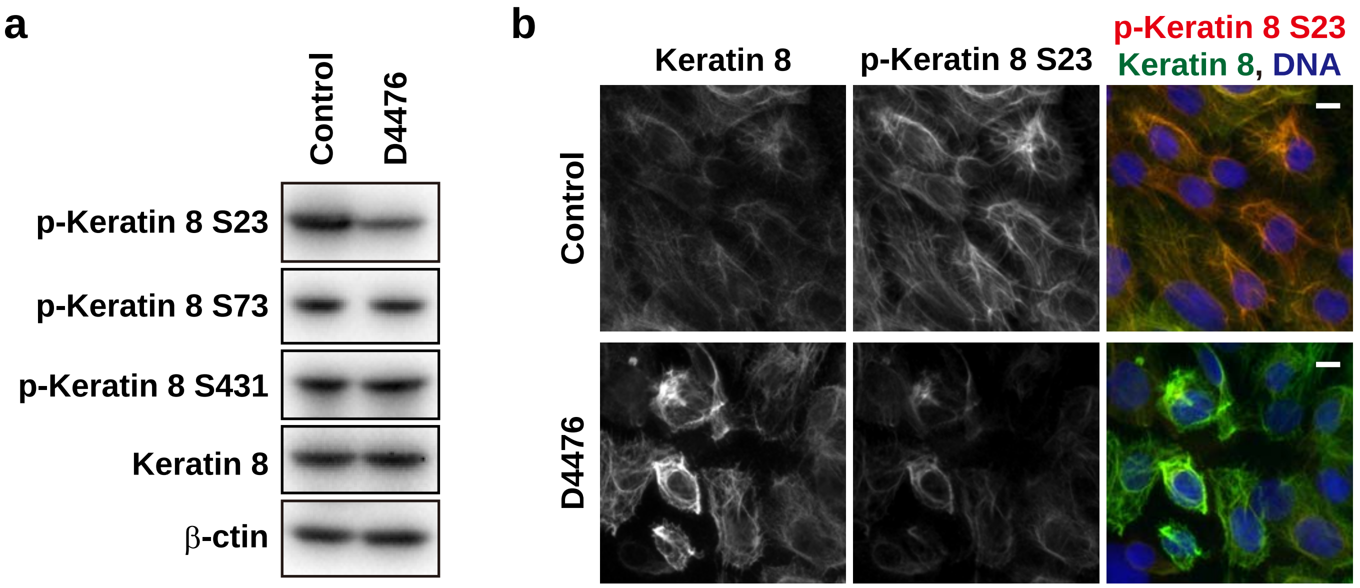
a
Control
D4476
p-Keratin 8 S23
p-Keratin 8 S73
p-Keratin 8 S431
Keratin 8
β-ctin
b
Keratin 8
p-Keratin 8 S23
p-Keratin 8 S23
Keratin 8, DNA
Control
D4476

Figure S8
